# Breeding polyploid *Populus*: progress and perspective

**DOI:** 10.48130/FR-2022-0004

**Published:** 2022-03-31

**Authors:** Xiangyang Kang, Hairong Wei

**Affiliations:** 1 Beijing Forestry Molecular Design and Breeding Advanced Innovation Center, National Engineering Laboratory of Forestry Breeding, Key Laboratory of Genetics and Breeding in Forest Trees and Ornamental Plants, Ministry of Education Beijing 100083, China; 2 College of Forest Resources and Environmental Science, Michigan Technological University, Houghton, MI 49931, USA

**Keywords:** *Populus*, Triploid, Tetraploid, Polyploid, Induction method, Parental selection, Regulatory mechanisms

## Abstract

*Populus* is a genus of 25−30 species of deciduous flowering plants in the family Salicaceae, which are primarily planted in short-rotation planations for producing timber, pulpwood, wooden products as well as bioenergy feedstock; they are also widely planted in agricultural fields and along roadsides as shelter forest belts for windbreak, decoration, and reduction of pollutants and noise. Moreover, their fast-growth and good adaptation to marginal lands enable them to provide some critical ecosystem services at various phytoremediation sites for land restoration and reclaimation. Thanks to their important roles, breeding for fast growing poplar trees has been one of the most important objectives for nearly a century. One of the most demonstrated, documented achievements in this aspect is polyploid breeding, especially triploid breeding. This paper critically reviews the various techniques used in inducing triploid plants, including natural 2n formation, artificial induction of 2n male and female gemmates through chemical or physical treatments, trait characterization of the triploid and tetraploid breeding populations, unveiling the molecular mechanisms underpinning the significantly improved traits, and identification and selection of the best triploid progenies. This review also recapitulated the challenges and strategies facing the future of triploid breeding in *Populus*, including amelioration of 2n gamete induction techniques and efficiency, selection of the best parents and identification of the best progrenies, utilization of the huge amount of genomic, transcriptomic, proteomic, metabolomic, and other omics data for selecting parents for improving target traits.

## Introduction

Tree polyploid breeding can involve the selection of naturally occurring polyploids and the artificial induction of polyploids. Natural polyploid plants have typically undergone long-term natural selection and adaptation, hence they exhibit strong genetic stability and adaptability. However, the number of naturally occurring polyploid plants is limited due to low fertility or infertility. As a result, identification of polyploid plants from a natural population is a great challenge for breeding and utilization. Moreover, although natural polyploids exhibit strong adaptability, their important economic traits may not meet human needs. As the technologies advance, and the advantages of polyploid plants, as well as the underlying mechanisms are unveiled, we will enter an era of artificial induction of polyploidy in plants.

According to Levan^[1,2]^, the plants that are particularly suitable for improvement via chromosome doubling are those that have relatively small numbers of chromosomes, large vegetative bodies as target traits, and are capable of cross-pollination. Later, Dewey added two more characteristics: perennial habit and vegetative reproduction^[3]^. Lewis^[4]^ maintained that reducing the dependence on seed production was important for the success of polyploid breeding as poplyploids, especially triploid plants, lack the ability to produce seeds. Compared with annual herbaceous plants, many forest trees can propagate vegetatively, and thus do not rely on reproduction of seeds for commercial utilization of polyploids. Furthermore, their perennial habits guarantee that new genotypes in which genetic gains are fixed can be utilized continuously for a long period after being successfully bred. Consequently, polyploid breeding of forest trees shows great potential for the development of new varieties especially fast-growing clones with short-rotation and high productivity.

Among forest trees, *Populus*, including 25−30 species, collectively called poplar and aspen, has been the subject of the most intensive research through polyploid breeding. The developmental stages for induction of chromosome doubling in microspores, megaspores, embryo sacs, zygotes, and somatic cells have been ascertained, and some real-time discrimination techniques for most appropriate time have been developed. A number of highly efficient systems for the induction of chromosome doubling in aforementioned tissues to cells have been established for generating triploids and tetraploids efficiently. Significant progress has also been made in understanding the trait variations in triploid and tetraploid poplar trees and their underlying formation mechanisms, resulting in theoretical and technical advances in the polyploid breeding of forest trees. In this review article, we will review these advances and discuss the challenges, strategies and perspective of polyploid breeding of poplars.

## Main methods for the induction of polyploidy in *Populus*

Nilsson-Ehle^[5]^ first found a giant triploid *P. tremula* in Sweden that had very large leaves and grew rapidly. This discovery brought widespread attention to using polyploid breeding and marked the beginning of poplar polyploid breeding. In the following seven decades, a series of new varieties of polyploids have been selected or bred and widely utilized in forestry^[6−11]^, which laid the foundation for the induction and utilization of polyploidy *Populus*.

### Breeding of triploid *Populus* by crossing natural 2n gametes with haploid gametes

**Figure 1:**
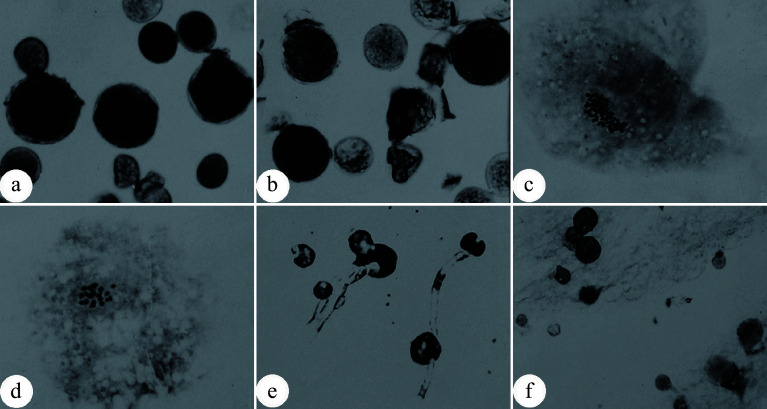
Natural occurrence of unreduced 2n pollens in *Populus tomentosa*. (a) Enlarged pollen grains produced by male clone B105 of *P. tomentosa*; (b) Enlarged pollen grains produced by male clone B111 *P. tomentosa*; (c) Chromosome number (n = 2x = 38) of an enlarged pollen; (d) Chromosome number (n = x = 19) of a normal pollen; (e) Germinated 2n pollens on medium; (f) Germinated 2n pollens on the stigma. (Figure source: References [9], [11])

Hybridization with natural 2n gametes is the most economical way to obtain triploids. Seitz first discovered that bisexual flowers of *P. canescens* (with partial anthers on the female inflorescences) could produce unreduced pollens; and self-pollination between the female portions of the bisexual flowers and the unreduced pollens yielded about 1% triploids^[12]^. Since then, 2n pollens have been found in *P. balsamifera*^[13]^, *P. canescens*^[14]^, and *P. tomentosa* (Fig. 1^[9,11]^). Triploids produced by crossing unreduced pollens with haploid female gametes showed much faster growth. However, to produce triploids by crossing haploid pollens with unreduced female 2n gametes encountered some challenges as the 2n female gametes could not be detected visually by morphological observation. The occurrence of 2n female gametes is generally indirectly determined by the appearance of triploid offsprings. Unquestionably, the other way to detect the 2n female gametes is to hybridize the 2n pollens with a female parent that may potentially produce gametes. For example, crossing of 2n pollens of *P. nigra* var. *italica* (Moench.) with *P*. × *euramericana* resulted in a tetraploid plant^[15]^. Natural 2n female gametes have later been found in the same way in *P. tomentosa*^[16]^.

In contrast to wide occurance of 2n pollen formation, natural 2n female gametogenesis in *Populus* appears to occur at lower frequencies, presumably because of the much smaller quantity compared to that of pollen. Unlike 2n pollen, natural 2n female gametes do not experience a competitive process for fertilization with haploid female gametes. Therefore, 2n female gametes are hard to acquire for triploid breeding despite their low occurrence rate.

Relative to the description of 2n gamete formation, fewer studies have focused on the cellular mechanism of 2n gamete formation in *Populus*. Previous studies have shown that 2n pollen formation in *P. tomentosa* may arise from the fusion of spindle poles in some cells, the disappearance of the phragmoplasts, or premature cytokinesis in meiosis II^[17−19]^.

### Generation of triploids by crossing artificially induced 2n pollens with haploid female gametes

**Figure 2:**
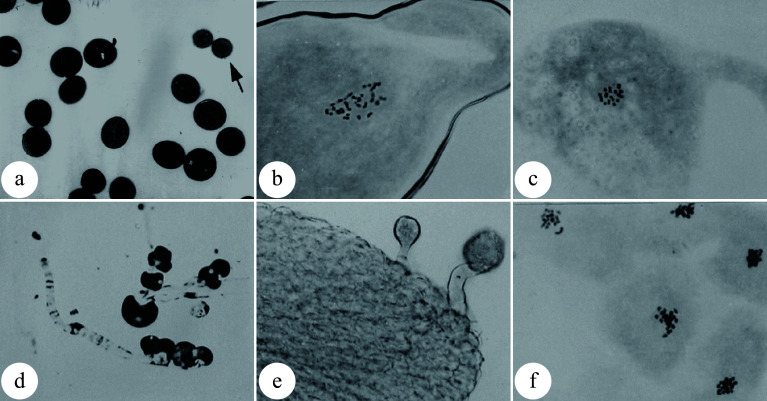
Pollen chromosome doubling of *P. tomentosa* × *P. bolleana* induced by colchicine. (a) 2n pollens induced by colchicine. The arrow indicates a normal pollen. (b) Chromosome number (n = 2x = 38) of an induced 2n pollen. (c) Chromosome number (n = x = 19) of a normal pollen. (d) Germination of induced 2n pollen on medium. (e) Germination of induced 2n pollens on the pistil stigma. (f) Chromosomes are aggregated into clusters after colchicine treatment. (Figure source: References [9], [23])

To improve the efficiency of 2n gamete formation and triploid breeding, artificial induction of chromosome doubling in microspores has been widely used to create polyploid plants. Johnsson and Ecklundh first applied colchicine to *P. tremula* and *P. tremuloides* male flower branches to obtain 2n pollen^[20]^; they then pollinated female inflorescences with the 2n pollen and successfully obtained triploid plants^[20]^. Since then, 2n pollen hs been induced in other species including *P. deltoides*^[20]^, *Pinus sylvestris*^[21]^, *P. balsamifera*^[21]^, *P. alba*^[22]^, *P. tomentosa* × *P. bolleana*^[23]^, *P. alba* × *P. glandulosa*^[24]^, *P. ussuriensis*^[25]^, *Populus canescens*^[26]^, *P. alba × P. tremula*^[21]^ and *P. pseudo-simonii*^[27]^ by chemical or physical treatments (Fig. 2^[9,23]^) for generating triploids. The most efficient chemical is colchicine with an optimal concentration of 0.2%−0.5%, whereas the most appropriate physical treatment is a high temperature in the range of 38−40 °C^[9,21,23−26,28,29]^. Compared with chemical induction, physical induction is a simple and inexpensive approach and thus enables batch treatment of more plant materials at one time. However, it is difficult to ensure the production of high quality 2n pollen when male flower branches are cultured in tap water, and more efficient techniques that can yield higher quality of 2n pollens remain to be optimized due to unsynchronized developmental stages of flowers in the branches.

**Figure 3:**
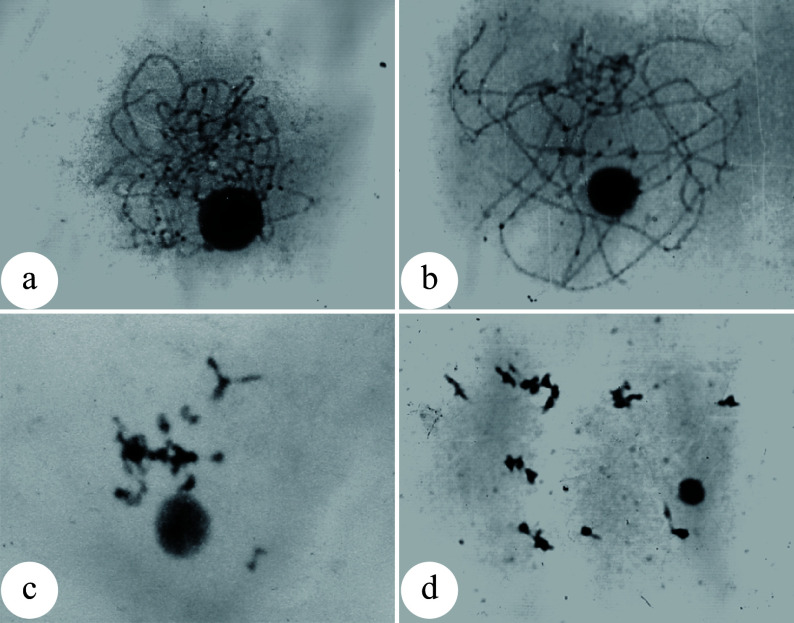
Meiotic stages of microspore mother cells in *P. tomentosa* × *P. bolleana*. (a) Leptotene; (b) Pachytene; (c) Diplotene; (d) Diakinesis. (Figure source: References [9], [23])

Understanding the cytokinetic processes and mechanisms can help to increase the induction efficiency of 2n pollen formation. First, it is critical to identify the sensitive stage for induction of 2n pollen. The meiotic pachytene of pollen mother cells (PMCs) was found to be the most sensitive stage to induce pollen chromosome doubling with colchicine. Likewise, the most effective phase to induce pollen chromosome doubling with high temperature is from the diakinesis to metaphase I phases of PMCs (Fig. 3^[9,23]^). More than 80% 2n pollen could be produced by these methods^[23,30]^. However, it is impossible to obtain 100% artificially induced 2n pollen owing to the asynchronous meiosis of PMCs. As a result, the induced 2n pollen are always mixed with regular haploid pollens. Compared with normal pollens, unreduced pollens show poorer fertilization competitiveness due to their stunted development, resulting in a low percentage of triploid plants after pollination with 2n pollen^[31]^.

Since haploid and diploid pollens have different sensitivities to ^60^C o-*γ*-ray radiation, a specific dose of ^60^C o-*γ*-ray can be applied to inhibit the development of haploid pollen. This kind of treatment results in an increase in the competitiveness of 2n pollen and results in as much as 3.8% of triploid plants^[32]^. Ewald and Ulrich also produced 0.27%−2.38% triploid poplars through microscopic selection of 2n pollens via *in vitro* pollination^[33]^. However, the generation rate of triploids through crossings with artificially inducted 2n pollen remains low because of the high competitiveness of haploid pollen, limiting the utility of this approach for triploid poplar breeding.

### Generation of triploids by chromosome doubling of female gametes

**Figure 4:**
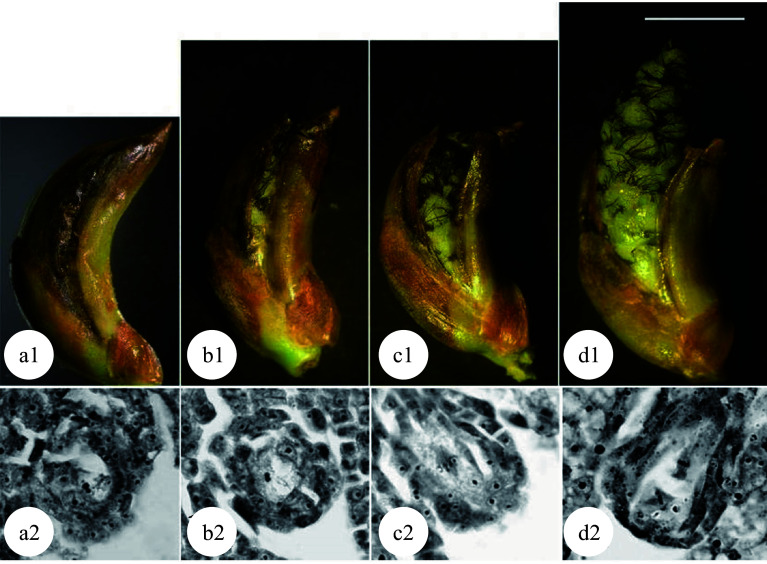
Meiosis of megaspore mother cells in 'Zheyin #3' and its relationship to female flower bud development. (a) In the pachytene stage, the flower buds swell, and the bud scales crack but are not yet open. (b) In the diplotene stage, the bud scales are slightly open, but the inflorescence is not exposed. (c) In metaphase I, the bud scales are open, and the inflorescence is slightly exposed. (d) In metaphase II, the bud scales are fully open one third of the inflorescence has emerged from the bud scales. Scale = 10 μm. (Figure source: Reference [38])

Compared to induced 2n pollen, induced 2n female gametes can be pollinated with normal pollen more easily to obtain triploid plants due to a lack of competitive process among 2n female gametes during pollination. Li et al. demonstrated this in *P. tomentosa* × *P. bolleana*^[34,35]^. After the female flower buds were treated with either colchicine or high temperature, 100% 2n female gametes, upon being pollinated with normal pollen, developed into triploids. Therefore, the induction of 2n female gametes rather than the pollination/crossing process is the most critical step for success. Mounting evidence indicates that the meiotic phase of megaspore mother cells is the most important factor that affects chromosome doubling. However, it is difficult to determine the exact meiotic phase of megaspore mother cells. In higher plants, the development of female buds is highly synchronized with male buds for the best pollination. Hence the most appropriate meiotic phase for inducing chromosome doubling of female gametes can be estimated by the developmental phase of male gametes^[36,37]^ or the developmental morphology of the female flower buds (Fig. 4^[38]^). The optimal treatment periods for chromosome doubling in megaspores are the pachytene to diplotene phases for colchicine treatment and the diplotene to metaphase I phases for high temperature treatment. As demonstrated in several previous studies, 16.7%−66.7% triploids can be obtained^[36−40]^.

Poplar embryo sac development follows the typical *polygonum*-type development process. Three rounds of mitosis are required for a megaspore to develop into the mature embryo sac. Based on this fact, 2n female gametes can be induced by physical or chemical treatment during the embryo sac development. Kang et al. used *P. alba* as the female parent and *P. tomentosa* as the male parent, and treated the female inflorescence for 24−36 h after pollination with 0.2% colchicine solution and obtained 57.1% triploid plants^[41]^. The optimal treatment stage for chromosome doubling of the embryo sac in 'Zheyin #3' was at the four-nucleate stage^[42,43]^. The highest triploid generation rate of 66.7% was achieved when colchicine treatment was conducted for 54−66 h after pollination^[43]^. When high temperature treatment was applied for 66−72 h after pollination, the highest triploid generation rate was 83.3%^[38,39,44]^.

### Generation of tetraploids by somatic or zygotic chromosome doubling

Many studies have investigated chromosome doubling in somatic or zygote cells in poplar, mainly by applying physical or chemical treatments to terminal buds^[45]^, seeds^[46]^, developing embryo sac^[37]^, calli^[47]^, and zygotes^[48,49]^ to induce tetraploid plants^[22,45−50]^. Adventitious buds cultured *in vitro* originated from single cells^[51]^. Therefore, when adventitious bud primordial cells are formed, physical or chemical treatments can effectively induce chromosome doubling while avoiding the production of chimeras, making it an ideal means for the induction of chromosome doubling in *in vitro* somatic cells^[52]^. The optimal treatment conditions for chromosome doubling in somatic cells differed among genotypes of *P. cathayana* hybrid seedlings^[53]^. Factors such as the pre-culture duration, colchicine concentration, and exposure time have significant effects on tetraploid induction rate in *in vitro* culture^[52,53]^. When a thin layer of callus cells begins to form on the incision of explants such as *in vitro* cultured leaves, 7.9%−13.2% tetraploids can be produced by colchicine treatment^[54]^.

**Figure 5:**
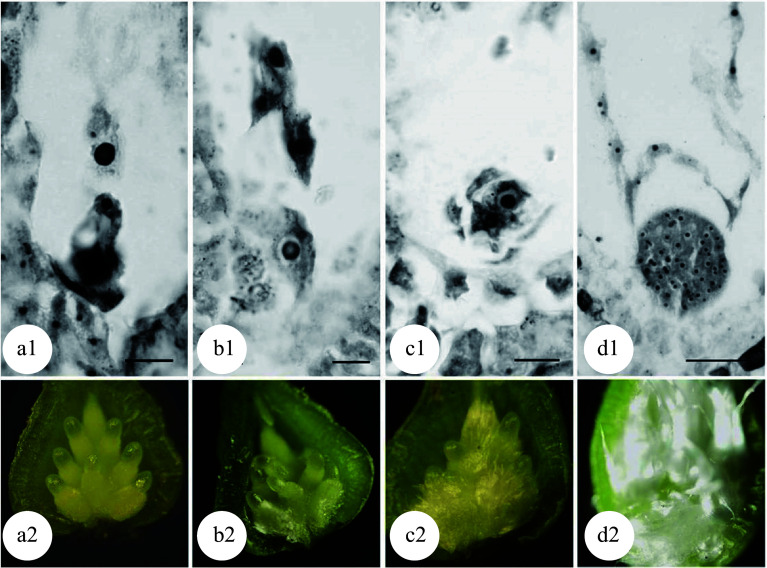
Developmental stages of zygote and flocculent fibers in the ovary of 'Zheyin #3' × *P. beijingensis.* (a) During double fertilization, flocculent fibers occur around the funicle in the ovary, but they are not visible to the naked eye. (b) The dormant zygote forms after fertilization, and the flocculent fibers cover the surface of the funicle and are visible to the naked eye. (c) In the two-celled embryo stage, the flocculent fibers begin to wrap the ovule but do not fill the ovary. (d) In the globular embryo stage, flocculent fibers completely wrap the ovule and fill the ovary. Scale = 10 μm. (Figure source: Referencecs [49])

Chromosome doubling can also be induced after pollination in zygote cells. Because a zygote is a fertilized egg cell, all tetraploid plants formed are non-chimeras. Mashkina et al. obtained tetraploid plants in *P. alba* by treating zygotes with colchicine at the first mitosis stage^[22]^. However, the optimal treatment stages for the chromosome doubling in zygote cells remains unknown. By treating large amounts of plant materials, most studies resulted in poor repeatability and low induction efficiency. A correlation has been reported between the development of flocculent fibers (seed hairs) in the poplar ovary and the development of the zygote^[48,49]^. When the flocculent fibers in the ovary begin to wrap the middle and lower parts of the ovule but have not yet completely covered it (Fig. 5^[49]^), physical or chemical treatments effectively induced zygote chromosome doubling in *P. adenopoda* and a (*P. pseudo-simonii* × *P. nigra* 'Zheyin #3') × (*P*. × *beijingensis*) hybrid, and obtained as high as 14.1%^[48]^ and 7.41%^[49]^ tetraploid plants, respectively.

### The methods for induction of 2n gametes and their genetic differences

There are three main methods of breeding triploids based on 2n gamete induction: 1) first division restitution (FDR)^[55]^, the FDR gametes comprise the non-sister chromatids of each homologous chromosome (FDR); 2) second division restitution^[56]^, FDR gametes comprise of the sister chromatids of each homologous chromosome (SDR); and 3) post-meiotic restitution gamete (PMR), meiotically formed haploid spores undergo an extra round of genome duplication, and consequently yield fully homozygous 2n gametes^[57]^. Theoretically, FDR-type and SDR-type 2n gametes contain 70%–80% and 30%–40% of the heterozygosity of their parents, respectively^[58,59]^. For example, the heterozygosity of FDR-, SDR-, and PMR-type 2n gametes from the same *P. cathayana* parent combination were 74.8%, 39.6%, and 35.9%, respectively^[60]^. Three triploid populations produced by these three methods also differ in growth, photosynthetic, and physiological traits. The highest average values of growth traits were observed in the FDR-type triploid population. Although the highest average values of growth traits for the SDR-type and the PMR-type triploid population, there were also some excellent genotypes^[61]^. This is because regions with high transcript abundance have higher frequencies of homologous recombination^[62]^.

## Trait variations of poplar polyploids and the underlying molecular mechanisms

### Trait characteristics and variations in poplar polyploids

**Figure 6:**
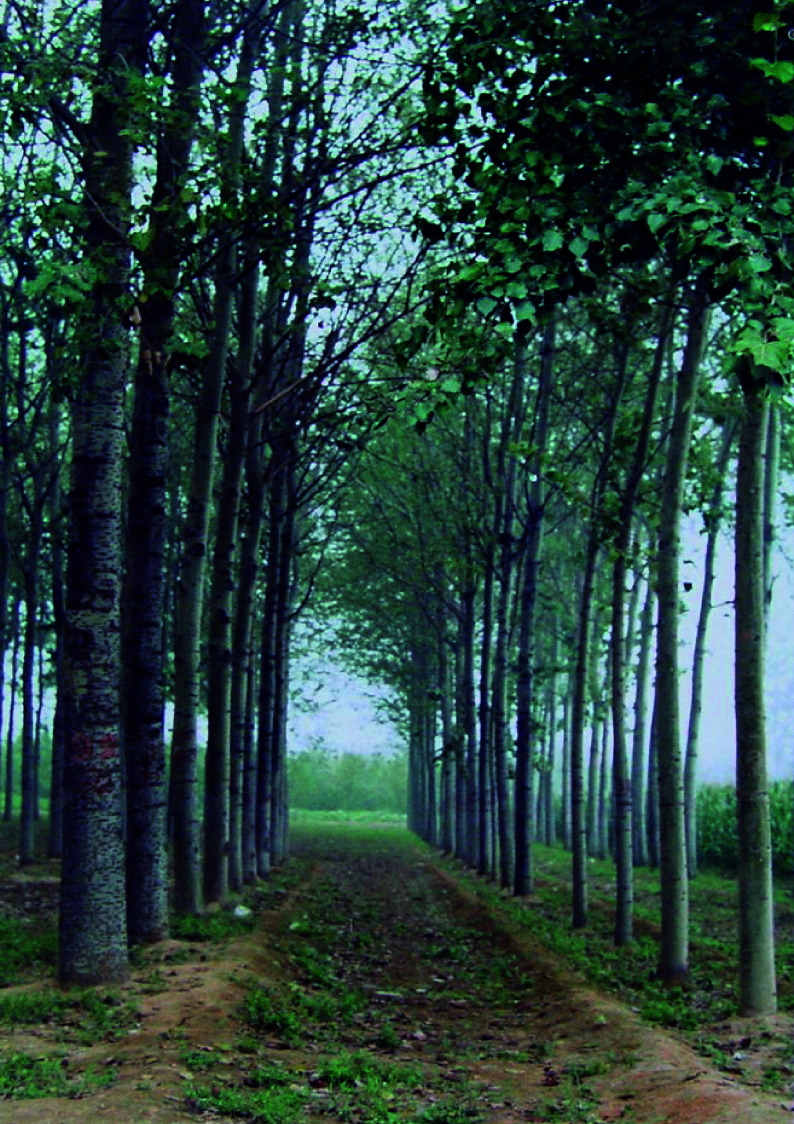
Eight-year-old triploid *P. tomentosa* trees show significant advantages in growth over diploid clones (not the same parents). Triploids are on the left, and diploids are on the right.

Height and diameter are the two most prominent traits that significantly increase in triploid poplars. Nilsson-Ehle found tree height, diameter at breast height (DBH), and overall volume of natural triploid *P. tremula* were 11%, 10%, and 36% higher than those of diploids, respectively^[63]^. The growth in height and DBH of triploid *P. tremuloides *x *P. tremula* hybrids were at least 20% higher than those of same-aged diploids^[6,7,64]^. The stem volume of the triploid clone B301 of *P. tomentosa* was 3.5 times that of its diploid parent^[11]^ (Fig. 6).

All elite varieties of poplars widely cultivated in China, such as *P*. × *canadensis* 'I-214', *P*. × *euramericana* 'Zhonglin-46', *P*. × *langfangensis*-3, and *P. alba* × *P. berolinensis*, are triploids that were bred by selection from thousands of hybrid offspring^[8,10]^. They all exhibited remarkable advantage in vegetative growth.

Larger sizes of cells and organs are clearly shown in triploid poplars. Larger tree stems and leaves were first indicators of natural polyploid poplars^[63]^. The widths of leaves of one-year-old seedlings in triploid *P. tomentosa* reached 53 cm^[9]^. Leaf areas of most individuals of the triploid population obtained by sexual polyploidization exhibit much greater than those of the diploid individuals derived from the same parents^[9]^, but some exceptions of triploid plants with leaf areas smaller than diploid plants are also observed. Greater leaf sizes increase the photosynthetic areas and promote the vegetative growth of triploids. Triploids also have longer fibers. For example, the average fiber length of a triploid hybrid of *P. tremula* × *P. deltoides* is 18% longer than that of the diploid control^[6]^. The average fiber length of 5-year-old triploid *P. tomentosa* is 1.28 mm, 52% longer than that of diploid *P. tomentosa* with the same age^[65]^; these triploid poplars also contain 17.9% lower lignin content and 5.8% more α-cellulose content^[65]^ as compared to diploids. The low lignin and high cellulose content of triploid poplars are due to the reduced number of fiber cells per unit volume, which reduces the cell surface area. Their rapid growth, long fibers, and low lignin content make triploid poplars excellent raw material for fiber industries such as pulping and papermaking.

There are significant differences in seedling growth and photosynthetic parameters among triploid hybrids produced using different chromosome doubling techniques and parental combinations. Triploid seedlings are significantly higher in average height, chlorophyll content, photosynthetic rate, and net photosynthetic rate of leaves at different positions, and larger in leaf area, diameter growth as compared with the diploid seedlings produced from the same parental lines. Moreover, there are greater variations in seedling growth, photosynthetic rate, leaf area, and chlorophyll fluorescence among different triploid individuals within than between populations^[9,61]^. This is likely because these growth-related traits are typically controlled by multiple genes and display a normal distribution in the population. This suggests that growth-related traits in triploid plants depend not only on the dose effect of chromosome doubling but also on the heterozygous effect conferred by the two distinct parents.

Although tetraploid poplars also have large cells, they generally grow slower compared with diploid and triploid poplars. Tetraploid *P. cathayana* has significantly larger guard cells but significantly slower height growth than the diploid counterparts^[9,54]^. It was found that the leaf area and photosynthetic rate at the tenth leaf position in tetraploid poplars are 0.17%−150.98% and 9.50%−57.53% higher than those of the diploid counterparts, respectively. However, after the formation of the tenth leaf, as the age increases, the area and net photosynthetic rate in the tetraploid plants beyond the tenth leaves are 16.07%−49.32% and 12.75%−48.66% lower than those of the diploid counterparts^[66]^. Overall, tetraploids grow slower than diploids because the total number of leaves is 75% less and the gross photosynthetic rate is 34.48% lower in tetraploid plants as compared to those in diploid plants^[66]^. In a few cases of hexaploid poplars produce by somatic chromosome doubling of triploids, they exhibit enlarged stomata, slower height growth, but shorter, thicker, and fewer roots^[67]^.

### The molecular mechanisms underlying trait formation in poplar polyploids

A considerable amount of research has been conducted to understand the genetic mechanisms that regulate the growth and development of polyploid plants. Microarray analysis and transcriptome sequencing (RNA-seq) have revealed a large number of differentially expressed genes (DEGs) in polyploids. Among the total DEGs, 5%−10% DEGs have expression levels significantly higher than those of the mid-parents value^[68−72]^; similar phenotypes were found in triploid rice^[73]^ and *Arabidopsis* tetraploids^[74]^. The circadian clock regulators, CCA1 and LHY, induce expression of TOC1, GI and downstream genes in *Arabidopsis* tetraploids during the daytime, augmenting the transcription of genes involved in photosynthesis, and starch metabolism. Although a great number of DEGs in the leaves of poplar triploids with fast growth or tetraploids with slower vegetative growth are identified, the number of circadian clock regulator-driven genes is not observed in DEGs of these polyploids^[66,75]^, indicating that the interactions among the more than three or four sets of chromosomes may obstruct normal gene expression.

Transcriptomic and proteomic studies in triploid poplars have revealed that the number of DEGs in terminal buds and young leaves are about 30 times greater than that of diploids^[76,77]^. These DEGs are mainly involved in metabolic processes, cell proliferation, DNA methylation, cell division, and meristem growth and development. In triploid poplar leaves, transcription factors (e.g., WRKY and MYB) and genes related to hormones (e.g., auxin and brassinosteroids) and growth (e.g., growth-regulting factor 5 (GRF5)) are significantly upregulated, leading to faster cell division and leaf growth associated with high photosynthetic capacity and photoassimilate accumulation in leaves^[75,78]^. Comparative proteomics studies on poplar triploids revealed that differentially expressed proteins (DEPs) in developing leaves are significantly enriched in the pathways related to photoreaction, photorespiration, the Calvin cycle, starch and sucrose metabolisms, the oxidative pentose phosphate (OPP) pathway, the tricarboxylic acid (TCA) cycle, nitrogen metabolism, and other major metabolic pathways and processes^[79]^. Wu et al.^[78]^ used a top-down graphical Gaussian model (GGM) algorithm^[80,81]^ to reconstruct the regulatory network-mediated by growth-regulating factor, GRF5-1^[78]^. This network operates in the leaf primordia and young leaves, and directly regulates leaf size and vegetative growth. Yeast one-hybrid screens (Y1H), electrophoretic mobility shift assays (EMSA), and dual luciferase analyses confirmed that GRF5-1 directly binds to the promoter of the cytokinin oxidase/dehydrogenase 1 gene, *CKX1*, and represses its expression^[78]^. Overexpression of *GRF5*-*1* in diploid poplar 84k produces larger leaves comparable to those of triploid poplar. Moreover, the zeatin and isopentenyl adenosine (IPA) content significantly escalate in the apical buds and the third-position leaves, implying the increase of leaf cytokinin content which in turn results in larger leaves, higher photosynthetic rates, and faster growth^[78]^. Since *GRF5*-*1* is a gene that is selectively boosted in poplar triploids, the above results suggest that selective activation of some growth-regulators is one mechanism underlying the advantage of vegetative growth in triploid poplars^[78]^.

**Figure 7:**
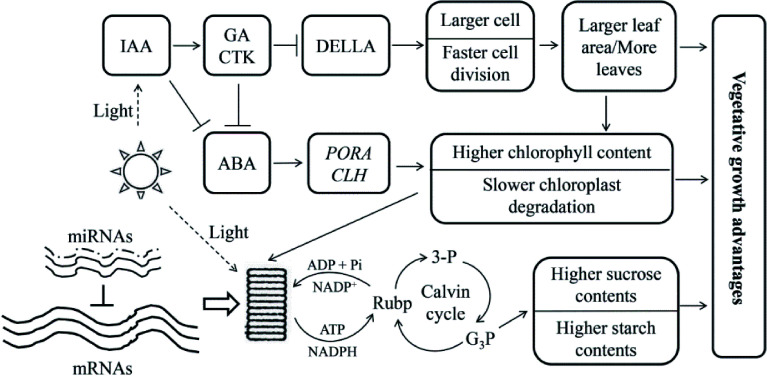
Genetic regulation model of allotriploid *P. cathayana* with enhanced vegetative growth. Arrows indicate promotion, and short lines indicate inhibition. The three solid lines next to 'mRNAs' indicate the dose effect in the triploid, and the dotted lines next to 'miRNAs' indicate the lack of a dose effect. (Figure source: Reference [82])

Transcript abundances are not always consistent with the abundance of their corresponding proteins in triploid poplar leaves, indicating that the traits changed may, to some degree, be regulated by post-transcriptional regulation^[79]^. RNA-seq studies of DEGs in triploid *P. cathayana* leaves showed that, compared with diploids, allotriploid *P. cathayana* showed significant changes of genes involved in vegetative growth^[82]^. However, miRNAs related to vegetative growth traits in allotriploids are expressed at similar levels to those in diploids during leaf development^[83]^ and no obvious dose effect is observed in vegetative growth-related miRNA expression in triploid leaves. These results imply that transcription and post-transcriptionally may be syncronized to empower greater vegetative growth in allotriploid *P. cathayana* (Fig. 7^[82]^).

**Figure 8:**
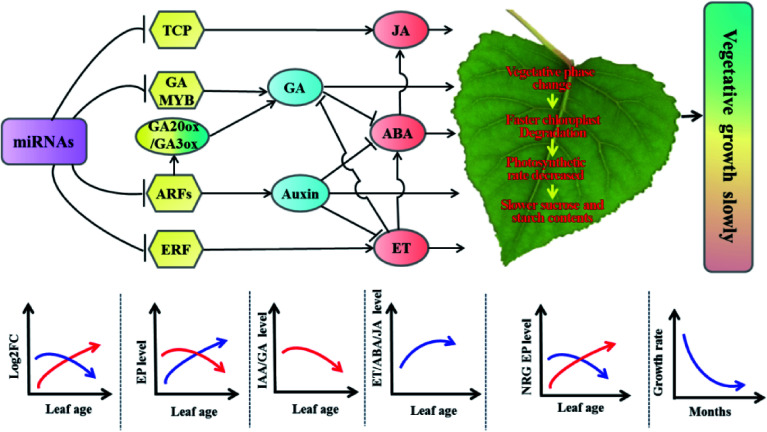
Molecular regulatory model of tetraploid poplars with slower vegetative growth than triploid poplars. Arrows indicate promotion, and short lines indicate inhibition. The red curves represent the expression of differentially expressed miRNAs positively associated with tetraploid vegetative growth or DEGs positively associated with vegetative growth. The blue curves represent the expression of differentially expressed miRNAs or DEGs negatively associated with tetraploid vegetative growth. ET, ethylene; EP: expressed protein; NRG, negative related growth; ABA: Abscisic acid; JA: asmonic acid; GA: gibberellic acid; FC: fold change. (Figure source: Reference [66])

To understand the slower-growing tetraploid poplars, RNA-seq and miRNA-seq were performed and the results revealed a dosage effect of miRNA expression on leaf growth in tetraploid poplar^[53,66]^. The differentially expressed miRNAs whose target genes are positively, and negatively correlated with vegetative growth manifest a gradually increased and decreased expression, respectively. These changes resulted in the suppression of vegetative growth-related gene expression, ultimately causing slower vegetative growth in the tetraploids (Fig. 8^[66]^). These results are consistent with the genetic regulatory model of allotriploid *P. cathayana* with enhanced vegetative growth^[75]^.

## Problems and effective stratagies for higher efficiency polyploid breeding

Polyploid breeding in poplars has proven that triploids consistently show vegetative growth advantages over either diploid counterparts (e.g., with same parents) or unrelated artificially selected diploid trees (e.g., elite clones). As a result, triploid breeding has become the the major focus of poplar research and and an effective approach to increase vegetative growth and wood productivity. Multiple trait improvements has been achieved through a single round of breeding, resulting in new poplar varieties with fast growth, long fibers, low lignin but high fiber content that are suitable for short-rotation fiber industries. In the past two decades, empherical protocols in chromosome doubling techniques for megaspores, embryo sacs, zygotes, and somatic cells have been developed, enabling the rapid and efficient production of triploid poplars. Nevertheless, some problems and challenges still exist, and the underlying mechanisms of polyploid breeding remain fragmented, and more technical details need to be developed. To achieve the goals of genetic improvement, future research plans and strategies may need to be refocused on the following aspects:

Firstly, despite the fact that polyploid induction through chromosome doubling can be achieved, the efficiency remains low and less predictable. Although the gamete chromosome doubling induced via physical condition or chemical regeants has shown to yield significantly improved growth traits of triploids^[9,40,44]^, the more efficient and predictable protocols with different chromosome doubling techniques must be developed to obtain a large number of polyploid varieties with significant heterosis in various target traits of interest.

Secondly, the construction of breeding populations and the selection of hybrid parents for polyploid poplar breeding are critically important for further improvement of the breeding efficiency. Present studies have shown that polyploid plants seem to rely on heterozygosity to generate the heterosis^[84]^. In the polyploids that were acquired by physical or chemical treatments of parents to induce 2n gametes, the traits such as growth of the triploid offspring show great differences^[9,40,44]^. In full-sib populations derived from different combinations of *P. tomentosa* parents that were crossed without any physical or chemical treatment, some hybridization offspring were triploids. The triploids of the best parental combinations exhibited distinct growth advantage in diameter and height^[16,85]^. To fully realize the benefits of triploid poplar breeding, we should emphasize the construction of the breeding population and the selection of elite parents for polyploid breeding. For example, we can take the advantage of SSR genetic distance information, and elite paternal parent selection and genetic assessment to construct more effective breeding populations, and then produce new triploid poplar varieties with superior growth traits through gamete chromosome doubling. The ploidy advantages derived from chromosome doubling and the heterosis generated by the combination of distinct parents can thus be used more effectively^[85,86]^. Furthermore, genetic, transcriptomic, proteomic and metablomic information can be integrated to assess and accelerate construction of breeding populations and selection of hybrid parent pairs that can generate high heterosis.

Thirdly, besides parental selection, the influence of different gamete induction methods on the efficiency of polyploid breeding should be taken into account as the three 2n gamete induction methods, FDR, SDR and PMR, can generate gametes with distinct heterozygosity^[62,87,88]^. More attention should be paid to FDR 2n gametes in practical work as they possess a relatively higher heterozygosity and can thus more efficiently transfer parental heterozygosity and epistasis to the progeny. Nevertheless, SDR- or PMR-type 2n gametes may be more suitable for triploid breeding if tree species exhibit poor synchrony in meiosis I during gametogenesis.

Finally, unveiling the genetic regulatory mechanisms of vegetative growth traits in polyploid poplars is also critically important for designing effective breeding plans and strategies and achieving the highest genetic gains on various traits. Previous studies have revealed that gene expression of polyploid poplars involves post-transcriptional regulation. For instance, miRNA expression levels determine the expression levels of their target genes and associated traits^[66,75]^. However, it remains unclear why the miRNA expression in tetraploids shows an obvious dosage effect during leaf development, whereas the miRNA expression in triploids does not. Studies also show that polyploidization can lead to changes in DNA methylation, thereby impacting gene expression^[89−91]^. The DNA methylation level of terminal buds in triploid poplars was found to be lower than that of diploid poplars based on methylation-sensitive amplification polymorphism (MSAP) markers^[92]^. Several questions then arise: Is the differential expression of miRNAs associated with these changes in DNA methylation after chromosome doubling in polyploid poplars? If so, how does DNA methylation regulate the expression of miRNAs and their target genes after polyploidization? Further investigation of variation in DNA methylation patterns and their correlation with gene expression and post-transcriptional regulation may help us to better understand the genetic control of vegetative growth in plants with different somatic ploidy levels and may enable us to design more effective research plans for poplar polyploid breeding.

Due to their large body sizes, long juvenile periods, and long lifespans of forest trees, it is inappropriate to use crop breeding strategies to achieve high levels of heterosis in trees. Strategies that are specifically suitable for poplar breeding must be developed. The superior performance of polyploid poplars produced by sexual polyploidization provides a new approach for the rapid development of poplar breeding. In light of global climate change and increasing lumber shortages, polyploid breeding of poplars is likely to become more practical as the awareness of its high value and development of new technologies and methods continues. Polyploid breeding will play an important role in breeding new poplar varieties with high-quality, fast-growth and stress tolerance.
